# Editorial: Multidimensional approaches to suicide prevention: innovations, challenges, and future directions

**DOI:** 10.3389/fpubh.2026.1820857

**Published:** 2026-03-23

**Authors:** Eduardo Fernández-Jiménez, Allyson Kelley

**Affiliations:** 1 Faculty of Law, Education and Humanities, Universidad Europea de Madrid, Madrid, Spain; 2 Department of Child and Adolescent Psychiatry, Clinical Psychology and Mental Health, La Paz University Hospital, Madrid, Spain; 3 Hospital La Paz Institute for Health Research (IdiPAZ), Madrid, Spain; 4 Allyson Kelley & Associates PLLC, Sisters, OR, United States

**Keywords:** challenges, editorial, innovations, prevention, suicide, structural and social determinants of health

Suicide remains one of the most devastating and complex global public health challenges that also requires public health actions (1). The annual rate of suicides worldwide is estimated to be 9.4 per 100,000 people (95% CI: 8.5–10.3), with higher rates in males (13.3) than females (5.7) (2). Furthermore, suicide has emerged as the third leading cause of death among individuals aged 15–29 years. Notably, 73 percent of global suicides occur in low- and middle-income countries (1). Suicidal behavior results from a complex interaction of various factors (biological, clinical, psychological, and socio-cultural), as outlined by the following multidimensional three-level model: (a) *distal-predisposing factors*: genetic and neurobiological vulnerability, early life adversity, and personality traits; (b) *mediating factors*: chronic mental disorders, substance use, and persistent interpersonal difficulties; and (c) *proximal-precipitating factors*: relationship breakdown, job loss, access to lethal means, exposure to suicide in others, and acute mental health symptoms (3).

Accordingly, this Research Topic, titled “*Multidimensional approaches to suicide prevention: innovations, challenges, and future directions*” encompasses 22 contributions that address the intricate interplay of cultural, social, economic, psychological, and biological factors in suicidal behavior while highlighting innovative approaches for assessment and intervention.

## Macrosystemic-level analysis: cultural, social and economic determinants

A critical dimension of suicide risk is the influence of macrosystemic factors. In particular, a global analysis of 183 countries conducted by Lyu et al. revealed that while global economic indicators, such as GDP per capita, are generally negatively correlated with suicide rates, these relationships shifted significantly when stratification analyses were conducted according to income groups among nations. Specifically, in high-income countries, these indicators may show positive correlations, probably because of increased social pressure and work competition, resulting in more mental health problems and an increased suicide risk. In this sense, the impact of economic shocks is further evidenced by the study of Timming, conducted among Australian men during the COVID-19 pandemic, finding that those who experienced job loss were 2.77 times more likely to report suicidal ideation at that time.

Beyond purely financial metrics, social empowerment may be a strong preventive factor. In particular, a quasi-experimental study carried out by Arvate et al. in Brazil revealed that municipalities with female mayors experienced a reduction in suicide rates among married women. This finding suggests that female role models can effectively challenge oppressive social norms. Conversely, Cahyono et al.'s study, conducted in rural agrarian contexts in Indonesia, highlights how micro-level vulnerabilities are shaped by environmental and social structures through the interaction between seasonal stress, such as crop failure during dry seasons, and cultural myths (e.g., *Pulung Gantung*: when a glowing red or yellow light, like a comet, is seen falling onto a house or area, it is thought to be a sign that someone living there might commit suicide, often by hanging).

## Individual-level analysis: integration of biological markers with clinical and psychological domains

Improving suicide risk assessment requires moving beyond subjective clinical interviews to objective and quantifiable supplementary markers. In this sense, in the study of Yiming et al. on patients with major depressive disorder (MDD), a significant non-linear association was observed between somatic symptom burden and suicidal ideation, with a critical risk threshold identified at a total score of 49 on the Somatic Symptoms Inventory (SSI). Moreover, metabolic health emerges as a key factor, which was observed in Zhang, Zhang, and Hang's study, in which the visceral adiposity index (VAI) showed a threshold effect on suicidal ideation, partially mediated by fasting blood glucose levels, principally among older adults, women, and patients with diabetes or hypertension.

Also, in specific populations, researchers are refining predictive tools through symptom-level analyses. In particular, a cross-lagged network analysis conducted by Zhang, Zhang, Hu et al. among undergraduate medical students in China identified insomnia symptoms, specifically daytime dysfunction and decreased well-being during the day, as pivotal nodes that predicted suicidal ideation factors within a bidirectional relationship. Additionally, among patients with thyroid cancer, a newly developed and validated nomogram proposed by Zhou et al. incorporates seven quantifiable risk factors for suicide, including histologic type and radiation therapy, to enable the early identification of high-risk survivors. Similarly, as Xu et al.'s review states, late-life depression (LLD) is increasingly considered distinct from adult depression, requiring specific assessment instruments, such as the Geriatric Suicide Ideation Scale (GSIS), which covers suicidal intent, hopelessness, social support, and physical health domains.

## Special consideration for vulnerable populations and epidemiological trends

This Research Topic sheds light on often-overlooked populations. For example, according to Jiayi et al.'s study, Chinese migrant workers face an exacerbation effect due to cumulative risk, in such a way that experiencing four or more adverse childhood or adulthood experiences (such as community violence or workplace discrimination) leads to a rapid increase in self-harm and suicidality. Additionally, among individuals who have experienced the loss of a loved one to suicide, Liang et al. found that chronic negative life events and an additive interaction between depression and hopelessness significantly increased the risk of developing suicidal ideation. Additionally, the study by García-Jarquín et al. concluded that the use of technologies, social isolation, depressive symptomatology, and repeated exposure to traumatic experiences increased suicidal ideation among students. Finally, Musgrave and Lamis highlighted that popular musicians are an at-risk occupational group for suicide and pointed out the lack of suicide prevention strategies for this specific population.

Additionally, retrospective studies of emergency departments, such as that of Yang et al., provide interesting and relevant epidemiological information. In this sense, ambulance records in China from 2018 to 2022 indicated that drug poisoning was the predominant method of suicide, a trend observed across all age groups and with a particular prevalence among younger individuals. In Australia, a longitudinal study by Mnatzaganian et al. on regional hospitalizations revealed that self-harm cases have become significantly more complicated over the last decade, with major complexity admissions rising from 9.3% to 43.5%. Importantly, Wang et al. identified that suicide cases, of hospitalized patients with mental disorders, accounted for 32.5% of all medical malpractice cases in comprehensive and tertiary hospitals in economically developed regions in China. This was attributed, among other factors, to significant deficiencies in the specialized care protocols.

## Innovative interventions and educational strategies

A paradigm shift is underway from reactive management to proactive, recovery-based prevention. In this sense, the “recovery-based model,” proposed by Ramamurthy and Gregory, moves beyond the chronic illness model to target transdiagnostic risk factors such as emotion processing deficits, aiming for socio-occupational recovery for at least a year. In the community setting, Dash et al. (b) highlighted social prescribing as a novel avenue to address non-medical determinants of suicidal behavior, such as loneliness and social isolation. In this direction, the same authors, Dash et al. (a), proposed a model for Australia that emphasizes “warm referrals” (personalized hand-offs between care providers) to support follow-through among those at risk.

Notably, technology and cultural adaptation are central to modern interventions. The “Mind4Health” program, proposed by Caughlan et al., utilized culturally tailored text message interventions for American Indian and Alaska Native (AI/AN) youth, training the skills of “askable adults” to engage in mental health conversations with them. Further evaluation of the “Good Road of Life” curriculum, suggested by Small et al., highlighted the effectiveness of community-led, strengths-based training in fostering protective factors for AI/AN populations. Finally, the importance of professional training was underscored by a path model on Korean social work students conducted by Ko, who found that diverse educational experiences (classroom learning and field experiences) directly predicted the intention to perform gatekeeper roles by enhancing perceived preparedness and suicide-related knowledge.

## Conclusion

**Figure 1:**
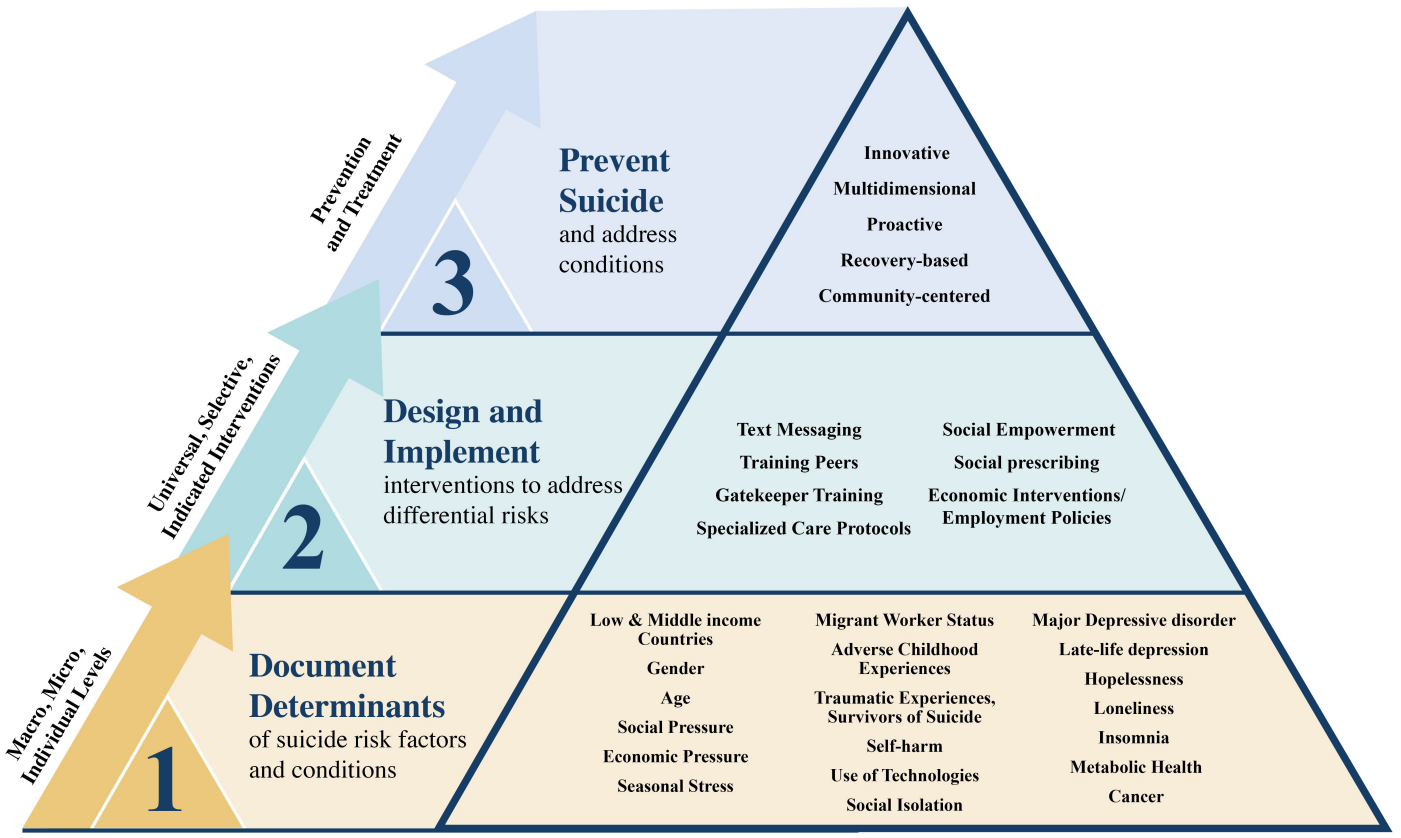
Synthesis of multidimensional approaches to suicide prevention Research Topic.

The twenty-two articles included in this Research Topic collectively provide a holistic and interdisciplinary framework for suicide prevention. We synthesized all these findings in Figure 1, following the model proposed by Kelley (4). The application of artificial intelligence and social network analysis to the integration of metabolic screening and social prescribing provides a roadmap for more effective, personalized, community-based, and culturally adapted prevention strategies. Importantly, the unique conditions, populations, and risk factors that contribute to suicide must be explored using various community-centered and empowerment-focused models. While randomized controlled trials and prospective designs are often used to document risks or develop interventions, they may not be effective or realistic in low- and middle-income countries. Efforts to build structural and systems-level changes while promoting multisectoral collaboration will be key to mitigating the global burden of suicide.

In light of the aforementioned considerations, developing national plans to address suicide, encompassing both prevention and postvention strategies, is imperative. These plans should incorporate a multicomponent approach that integrates three types of preventive interventions as follows (5): (1) *universal* (public education, lethal means restriction, and primary care physician training); (2) *selective* (targeting high-risk groups such as individuals with mental health problems, homelessness, or post-discharge from psychiatric care); and (3) *indicated* (focused on individuals with active suicidal behavior or recent attempts). Moreover, these national plans should be based on interdisciplinary teams that include members of the population most affected by suicide, clinical psychologists, psychiatrists, mental health nurses, social workers, occupational therapists, and other relevant professionals. However, to implement these plans effectively, the healthcare system must ensure the inclusion of an adequate number of mental health specialists and other professionals (6, 7). In this sense, suicide prevention initiatives should transcend traditional clinical settings and be embedded within community resources, where individuals grow and develop their lives naturally. Drawing on this emerging body of literature, it is imperative to develop international and nationally scaled strategies that adopt a multidimensional approach. This involves increasing efforts to tailor interventions for specific populations, emphasizing culturally and community-oriented strategies to address the underlying factors that differentially contribute to suicide risk within each demographic group. In conclusion, in response to this significant public health challenge, it is imperative to develop strategies that are globally informed and sensitive to local contexts.
